# Composite Membrane for Sodium Polysulfide Hybrid Redox Flow Batteries

**DOI:** 10.3390/membranes13080700

**Published:** 2023-07-27

**Authors:** Michelle L. Lehmann, Ethan C. Self, Tomonori Saito, Guang Yang

**Affiliations:** Chemical Sciences Division, Oak Ridge National Laboratory, Oak Ridge, TN 37831, USA

**Keywords:** redox flow battery, polysulfide, non-aqueous, electrochemical stability, Nafion, polypropylene

## Abstract

Non-aqueous redox flow batteries (NARFBs) using earth-abundant materials, such as sodium and sulfur, are promising long-duration energy storage technologies. NARFBs utilize organic solvents, which enable higher operating voltages and potentially higher energy densities compared with their aqueous counterparts. Despite exciting progress throughout the past decade, the lack of low-cost membranes with adequate ionic conductivity and selectivity remains as one of the major bottlenecks of NARFBs. Here, we developed a composite membrane composed of a thin (<25 µm) Na^+^-Nafion coating on a porous polypropylene scaffold. The composite membrane significantly improves the electrochemical stability of Na^+^-Nafion against sodium metal, exhibiting stable Na symmetric cell performance for over 2300 h, while Na^+^-Nafion shorted by 445 h. Additionally, the composite membrane demonstrates a higher room temperature storage modulus than the porous polypropylene scaffold and Na^+^-Nafion separately while maintaining high Na^+^ conductivity (0.24 mS/cm at 20 °C). Our method shows that a composite membrane utilizing Na^+^-Nafion is a promising approach for sodium-based hybrid redox flow batteries.

## 1. Introduction

**Figure 1:**
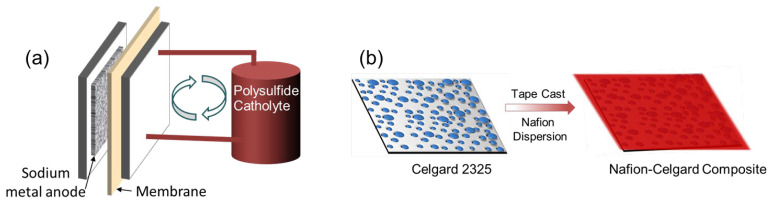
(**a**) Schematic illustration of a hybrid Na-polysulfide redox flow battery with a membrane separator. (**b**) Process of fabricating Nafion-filled Celgard 2325 composite membranes.

Long-duration energy storage (LDES) technologies are critical for enabling the widespread adoption of renewable energy sources such as wind and solar on the electric grid. Due to the intermittent nature of renewable energy sources, long-duration stationary energy storage systems are vital for providing a secure and stable electricity supply [1,2]. Redox flow batteries (RFBs) are well suited for such applications due to their unique advantage of having their energy density decoupled from their power density [3,4]. Non-aqueous RFBs (NARFBs) have emerged as a future alternative to aqueous RFBs (e.g., all-vanadium and Zn/Br systems), as they have the potential to use supporting electrolytes with wide operating voltage windows (>3 V) and a variety of redox couples [2,3]. One NARFB of interest is a hybrid RFB that utilizes an alkali metal anode, such as sodium, and a polysulfide catholyte (Figure 1a) [5,6,7]. The hybrid system allows for high specific energy density while utilizing low-cost, earth-abundant active materials. However, the realization of a hybrid NARFB for LDES applications requires the development of new membranes with robust mechanical properties, good (electro)chemical stability, high ionic conductivity, and low catholyte permeability.

Typical membranes for NARFBs include ionically conductive ceramics and polymers. Ceramic solid electrolytes (e.g., Na^+^ ß” Al_2_O_3_) eliminate the crossover of catholyte species but are typically only available in thick formats (e.g., 1 mm thick sheets) which are costly to manufacture. Compared to ceramics, polymer membranes can be fabricated using scalable roll-to-roll processes and are typically used as either a dense membrane, a porous separator, or a combination of both (composite). Dense membranes allow for high selectivity but generally have low ionic conductivity in nonaqueous electrolytes [8,9]. Porous polyolefin separators, such as Celgard, are chemically inert; however, the pore structure results in a significant crossover of active species [2]. On the other hand, composite membranes can contain a thin, dense ion-selective layer deposited on a porous support. Such a configuration can reduce area-specific resistance (ASR) while providing higher selectivity compared to the porous substrate.

Previously, we demonstrated that a thick Na^+^ cation exchanged Nafion membrane (120 μm) inhibited Na_2_S_x_ crossover, which enabled a hybrid Na|Na_2_S_x_ RFB with improved cycling stability and Coulombic efficiency compared to systems containing a porous separator [7]. Despite this promising performance, the prior study showed that Nafion adversely reacts with Na metal, forming a resistive passivation layer during extended cycling. This study reports the fabrication of a composite membrane (Na^+^-Nafion-Celgard), which combines the chemical stability of Celgard, with the high Na^+^ selectivity of Nafion. The composite membrane is expected to improve stability against Na metal while maintaining low catholyte permeability. The conductivity, polysulfide selectivity, and electrochemical stability of the composite membrane are compared to Celgard and a thin Nafion (25 μm) membrane. This study provides a simple and effective method for fabricating composite membranes for the advancement of high-performance non-aqueous energy storage technologies.

## 2. Experiments

### 2.1. Materials

Nafion dispersion (D-520, ≥1 meq/g) was purchased from BeanTown Chemical (Hudson, NH, USA). Celgard 2325 was purchased from Celgard (Charlotte, NC, USA), and Nafion 211 was purchased from the Fuel Cell Store (Bryan, TX, USA) and used without any purification steps. Diglyme (Sigma, Burlington, MA, USA, anhydrous, 99.5%) and sodium hexafluorophosphate (NaPF_6_, Sigma, 99.9%) were stored in the glovebox and used as received. The supporting electrolyte utilized for measurements (1 M NaPF_6_/diglyme) was dried over molecular sieves for at least 48 h before use. All other reagents were purchased from Sigma or Fisher Scientific (Hampton, NH, USA) and used as received.

### 2.2. Membrane Fabrication

Nafion dispersion was evaporated from the as-received water/1-propanol solution and made into a 20 wt% solution in dimethylacetamide. The solution was cast onto Celgard 2325 using a 50 mil gap doctor blade and dried on a hot plate at 50 °C. Nafion 211 (25 μm) and the composite membranes were exchanged to sodium form by soaking in a 1 M NaOH aqueous solution for 24 h. The solution was replaced with fresh NaOH three times. The membranes were rinsed well with deionized water and dried under vacuum at 80 °C for 24 h before being transferred to an Ar-filled glovebox.

### 2.3. Characterizations

Scanning electron microscope (SEM) micrographs were obtained by a cold-cathode field emission (FE) SEM system (Hitachi TM3030Plus Tabletop Microscope) at 15 kV accelerating voltage using secondary electron (SE) and back-scattered electron (BSE) mixed mode. The sample transfer time to the vacuum chamber of the SEM was less than 20 s. All infrared (IR) spectra were obtained from an FTIR spectrometer (Bruker, ALPHA, Billerica, MA, USA) using a diamond-attenuated total reflection (ATR) accessory. The wavenumber ranges from 3200 to 450 cm^−1^ with 32 scans in total. All IR measurements were performed in an argon-filled glove box with O_2_ and H_2_O < 0.1 ppm.

Dynamic mechanical analysis (DMA) was performed using a TA Instruments Q800 DMA (New Castle, DE, USA). Samples of a 9 × 15 mm size were measured in tensile mode at an operating frequency of 1 Hz and heated at 3 °C/min from 20 to 120 °C under a nitrogen atmosphere. Permeability measurements were conducted in a diffusion cell containing feed/permeate compartments separated by the membrane. 1 M NaPF_6_ in diglyme was added to both compartments, and the membranes were equilibrated overnight before Na_2_S_8_ in diglyme was added to obtain a 19.2 mM Na_2_S_8_ concentration on the feed side. Na_2_S_8_ crossover to the permeate was monitored by UV–vis spectroscopy at 293 nm by periodically removing an aliquot from the permeate solution. The removed aliquots were returned to the permeate solution within several minutes. The feed and permeate solutions were agitated using magnetic stirrers during the measurements. The permeability of the membranes was calculated using the following equation:

$$V\left(\frac{{dC}_{t}}{dt}\right)=A\frac{P}{L}(C_{0}-C_{t})$$

where *V* is the volume of solution on the feed side, *C*_0_ is the initial concentration of redox species on the feed side, *C_t_* is the concentration of the redox species on the feed side as a function of time, *A* is the effective area, *L* is the thickness of the membrane, and *P* the permeability of the membrane.

Samples for electrochemical measurements were prepared in an Ar-filled glovebox and equilibrated in a 1 M NaPF_6_/diglyme solution for at least 3 days. The thickness of Nafion 211 and the composite membrane increased after swelling in the electrolyte solution, to 31 μm and 57 μm, respectively. Membranes of a 15.9 cm diameter were sandwiched between 1.27 cm (diameter) sodium metal discs and sealed in a R2032 coin cell inside an Ar-filled glovebox. Sodium stripping/plating tests were performed at room temperature, except for the composite membrane, which was cycled at 30 °C for the first 800 h and then at room temperature for the remaining time. The cells were subjected to ±100 μA/cm^2^ for 0.5 h in each half cycle. The Na^+^ conductivity of the membranes was measured from 20 to 60 °C using AC impedance spectroscopy at open circuit by applying a 10 mV perturbation over the frequency range of 1 MHz–1 Hz. The resistance of the membranes was determined from the high-frequency intercept of the real axis. Electrochemical characterization was performed using a Biologic VMP3 potentiostat and EC-Lab^®^ software (Seyssinet-Pariset, France). Membrane ASR was determined using the following equation:

$$ASR=\frac{t}{\sigma }$$

where *t* is the membrane thickness and *σ* is the ionic conductivity measured by AC impedance spectroscopy.

Time-resolved AC impedance and open-circuit voltage measurements were performed using R2032 cells containing 0.25 m Na_2_S_8_ in 0.5 M NaPF_6_/diglyme as the catholyte, carbon paper (Sigracet 36AA, Fuel Cell Store) as the cathode current collector, and a sodium metal anode. The membrane (equilibrated in 1 M NaPF_6_/diglyme) was blotted to remove the excess electrolyte and then assembled in a cell containing catholyte-soaked carbon paper and sodium metal.

## 3. Results and Discussion

**Figure 2:**
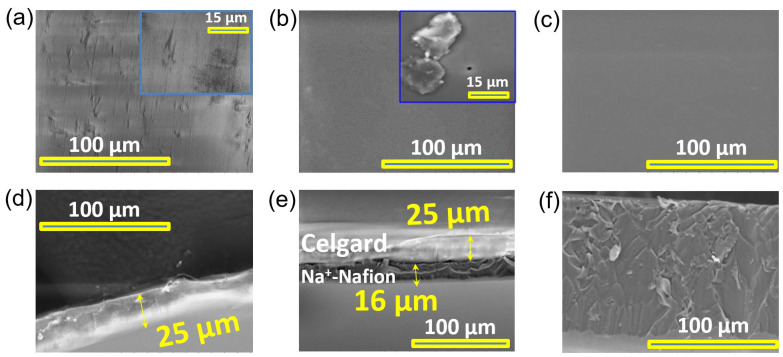
SEM micrographs of the plane view of (**a**) Celgard, (**b**) composite, and (**c**) Na^+^-Nafion 115. (**d**–**f**) The corresponding cross-section view of these samples in (**a**–**c**). Inset images for (**a**,**b**) represent a magnified view of the SEM image, with a scale bar of 15 µm.

A composite membrane (Na^+^-Nafion-Celgard) was fabricated by tape casting a Nafion dispersion (20 wt% solution in dimethylacetamide) onto Celgard 2325 followed by drying and conversion to Na^+^ form (Figure 1b). The membrane morphology was inspected by SEM as shown in Figure 2. The surface of Celgard has numerous micron-size defects and pores (Figure 2a). On the other hand, the composite membrane exhibits a much smoother surface (Figure 2b), similar to the Na^+^-Nafion benchmark (Figure 2c). However, a closer inspection indicates that pinholes exist in the composite membrane (inset of Figure 2b). The composite membrane exhibits preferential Na^+^-Nafion distribution on one side of Celgard (Figure 2e). Such a well-defined Na^+^-Nafion layer is preferable, as we expect a uniform Na^+^-Nafion layer to mitigate the sodium polysulfide crossover, while the Celgard-rich side can be placed against the sodium metal anode to improve interfacial compatibility. The thickness of the Na^+^-Nafion layer in the composite membrane was 16 µm, with the total thickness of the composite membrane being 41 µm (Figure 2e). The reduced thickness compared to Na^+^-Nafion 115 is expected to lower the composite membrane’s ASR.

**Figure 3:**
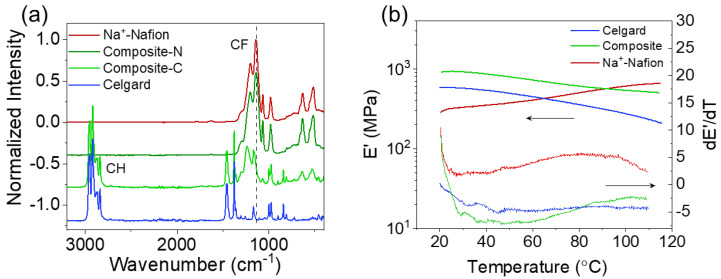
(**a**) IR spectra of Celgard, Na^+^-Nafion, and the two surfaces of the Na^+^-Nafion composite membrane. The Composite_C designation refers to the Celgard side of the membrane, while Composite_N designates the Na^+^-Nafion side of the membrane. (**b**) Thermo-mechanical properties of various membranes.

The distribution of Nafion on the composite membrane’s surface was also evaluated by FTIR spectroscopy (Figure 3a). The most distinguishable peak for Na^+^-Nafion is the C-F stretching mode centered at 1137 cm^−1^, whereas the peak at 2920 cm^−1^ features the C-H stretching for Celgard. There is no C-H stretching mode for the Composite-N (Na^+^-Nafion rich) of the composite membrane, indicating the presence of pure Na^+^-Nafion layer on that surface. On the other hand, Composite-C (Celgard rich side) exhibits both C-H and C-F stretching modes, indicating that Na^+^-Nafion and Celgard coexist on this surface. Notably, the C-F stretching mode blue-shifts to 1170 cm^−1^, suggesting that Nafion experiences a chain conformational change in Celgard’s pores.

The mechanical strength and thermal properties of the membranes were evaluated over the temperature range 20 to 120 °C by DMA (Figure 3b). The composite membrane exhibits a higher storage modulus (E’) compared to Celgard and Na^+^-Nafion 211 (Table 1). Celgard shows a decrease in the storage modulus with temperature, indicating a softening of the polymer backbone, but no thermal transitions occur, as the temperature remains below Celgard’s melting point (a blend of polyethylene and polypropylene, which melt at 130 and 160 °C, respectively) [10]. In comparison, Na^+^-Nafion’s storage modulus increases with temperature. No thermal transitions are observed for Na^+^-Nafion as the measurement temperature range is well below the glass transition temperature of Na^+^-Nafion (240 °C) [11]. The composite membrane’s storage modulus decreases with temperature, similar to Celgard, although the rate of change (-dE’/dT) decreases at temperatures > 90 °C due to the presence of Na^+^-Nafion (Figure 3b).

**Figure 4:**
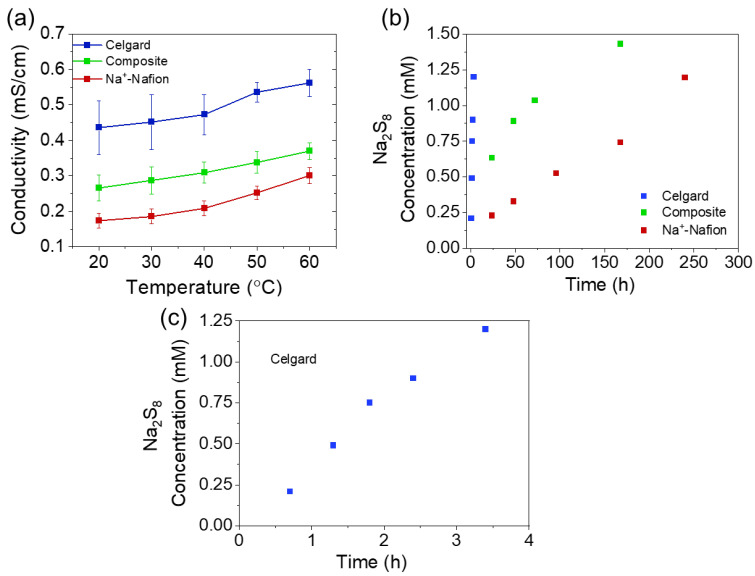
(**a**) Temperature dependencies of ionic conductivity for membranes soaked in 1 M NaPF_6_/diglyme. Error bars are the standard deviation of 3 replicates. (**b**) Permeate concentration of Na_2_S_8_ as a function of time for the various membranes. (**c**) Enlarged view of Figure 4b for the region pertinent to Celgard.

The Na^+^ conductivity of the membranes was measured by electrochemical impedance spectroscopy utilizing a sodium metal symmetric cell (Figure 4a). As expected, the Celgard separator exhibits the highest conductivity (0.44 mS/cm at room temperature) due to its porous structure, which allows for fast ion transport. The composite membrane shows slightly higher conductivity than Na^+^-Nafion (0.26 and 0.17 mS/cm, respectively). The conductivity of the composite membrane is similar to the expected value calculated from simple mixing, which considers the Nafion and Celgard layers resistances (and thicknesses) to be additive (0.22 mS/cm^2^). Importantly, the ASR of the composite and Na^+^-Nafion membranes (24 and 20 Ω cm^2^, respectively, Table 1) utilized in this work are significantly lower than the Nafion 115 membrane (88 Ω cm^2^) we previously reported in a Na|Nafion|Na_2_S_x_ hybrid RFB [7]. Notably, lower ASR is typically offset by the higher crossover rates of the catholyte redox active species.

We evaluated the permeability of Na_2_S_8_ through these membranes using a diffusion cell setup where the supporting electrolyte was 1 M NaPF_6_/Diglyme, and the feed contained 19.2 mM Na_2_S_8_. The Na_2_S_8_ concentration on the receiving side was monitored by UV–vis spectroscopy. As seen in Figure 4b, significant crossover of Na_2_S_8_ through Celgard occurs in <5 h, whereas similar Na_2_S_8_ permeate concentrations are only observed after several days for cells containing Na^+^-Nafion and composite membranes. The permeability of Na_2_S_8_ through Celgard is two orders of magnitude higher than that of the thin Na^+^-Nafion membrane (Table 1). The high permeability of Celgard is expected due to the membrane’s pore structure, which allows for the fast diffusion of redox species through the membrane. On the other hand, Nafion is a dense membrane, and redox species need to diffuse through the hydrophilic ion channels of the polymer [12,13]. The permeability of Na_2_S_8_ through the composite membrane is less than an order of magnitude higher than that of Na^+^-Nafion. The higher permeability of the composite membrane is due to the Nafion layer being slightly thinner than Nafion 211 and the small defects in the polymer coating on Celgard (Figure 2). In addition, solvent cast Nafion membranes have been found to exhibit higher permeability than commercial melt extrusion cast membranes due to larger ionic-cluster sizes [14].

**Figure 5:**
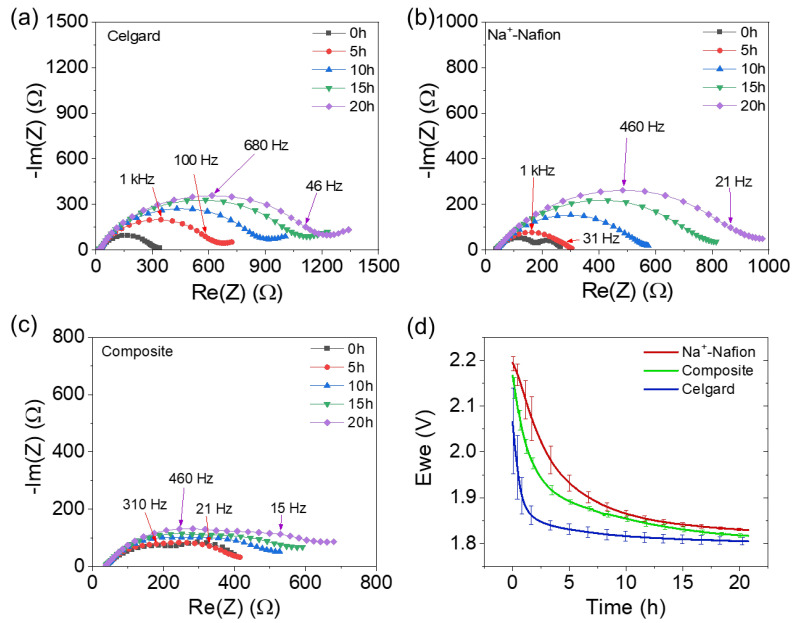
(**a**–**c**) Representative time-resolved electrochemical impedance spectra and (**d**) open-circuit voltage evolution of the Na|membrane|Na_2_S_x_ full cells. Error bars are the standard deviation of 3 replicates.

The evaluation of the electrochemical stability of each membrane was performed in a Na|membrane|Na_2_S_x_ coin cell configuration using time-resolved electrochemical impedance spectroscopy and open-circuit voltage measurements, as shown in Figure 5. The full cells were prepared with a sodium metal anode, membrane, and a 0.25 M Na_2_S_8_ in 0.5 M NaPF_6_/diglyme-soaked carbon paper. The Nyquist plots exhibit a high frequency resistance associated with the bulk membrane resistance. The low frequency response exhibits distorted, convoluted semi-circle(s), and may be due to any of the following processes: (i) passive films on the Na anode; (ii) charge transfer associated with the Na_2_S_8_ catholyte [15]; and/or (iii) the diffusion of redox active species with nonblocking boundary conditions. In this work, we do not attempt to precisely identify or decouple low-frequency features, and instead qualitatively assign them as interfacial resistance. In all cases, the interfacial resistance increases with time, indicating drift in the system (e.g., due to reactions at one or both electrodes). Plausible mechanisms whereby such drift may occur include: (i) the reductive decomposition of Na_2_S_8_ to Na_2_S on the Na metal surface due to catholyte crossover through the membrane; and (ii) the reductive decomposition of Na^+^-Nafion in contact with Na metal. In both cases, the unstable passivation of the Na anode is expected to increase the interfacial resistance with time. The initial interfacial resistance of the membranes is similar, with Nafion being the lowest (308 ± 35 Ω), followed by Celgard (360 ± 60 Ω) and the composite membrane (430 ± 33 Ω). However, the interfacial resistance increased quickly for both Celgard (1330 ± 210 Ω) and Nafion (1075 ± 105 Ω) samples over 20 h. In contrast, the composite membrane experiences the least increase in interfacial resistance among the three samples (750 ± 115 Ω). Similarly, the open-circuit voltage, E_we_, decays over time for all three membranes, mainly due to a self-discharge process in which Na_2_S_8_ crosses through the membrane and is reduced at the anode to form insoluble products. Na_2_S, which is insoluble in diglyme, is the expected thermodynamic endpoint for such reactions. The E_we_ value is the highest for the Na^+^-Nafion membrane after 2 h of relaxation, consistent with the permeability values mentioned above (Table 1). The initial E_we_ of Celgard is lower than that of Na^+^-Nafion and the composite membrane, further supporting the fast crossover of Na_2_S_8_ through the porous separator.

**Figure 6:**
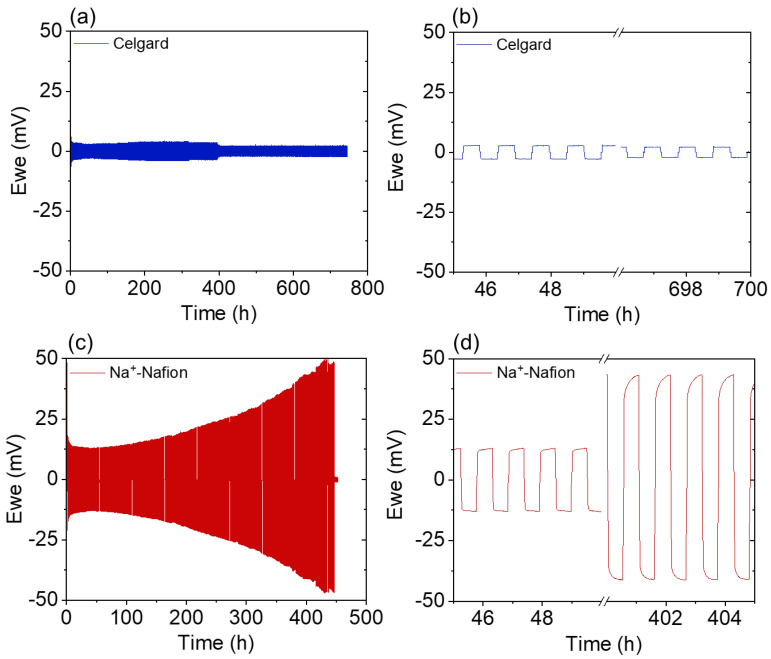

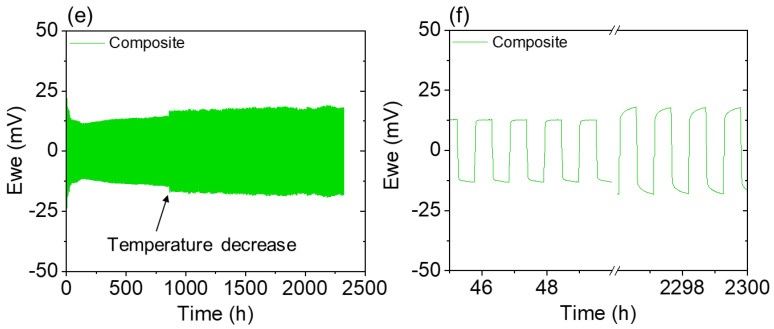
Sodium symmetric cell stripping/plating measurements for the various membranes, using 1 M NaPF_6_/diglyme electrolyte at a current density of 100 μA/cm^2^. Figures on the right are zoomed in sections of the figures on the left near the beginning and end of the cycling period. (**a**,**b**) Celgard, (**c**,**d**) Nafion, and (**e**,**f**) Composite. In (**e**), the temperature of the cell decreased from 30 °C to 20 °C at the point indicated.

The membranes’ electrochemical stability was further evaluated by sodium stripping and plating in a sodium symmetric cell at ±100 µA/cm^2^ (Figure 6). Celgard shows a stable cycling profile over the testing period of 750 h, suggesting that the diglyme-based electrolyte aids in stabilizing the sodium metal, as seen with previous works. A symmetric cell utilizing a Celgard separator and a NaBF_4_/diglyme electrolyte cycled at 100 μA/cm^2^ was reported to last for over 3000 h [16]. Comparably, symmetric cells utilizing carbonate-based electrolytes short circuit in <150 h [17,18,19], although it should be noted that the failure of symmetric cells is subject to testing protocol details (e.g., current density, areal capacity, cumulative capacity passed, etc.), which makes direct comparisons across studies difficult. Compared to Celgard, the cell containing a Na^+^-Nafion membrane shows a steady increase in the overpotential with time before shorting at 445 h. The increase in overpotential indicates that Nafion is not electrochemically stable against sodium metal and that a resistive solid-electrolyte interface layer is formed [20], which is consistent with the AC impedance measurements shown in Figure 5. Even so, the Na^+^-Nafion membrane outperformed linear and crosslinked poly(ethylene oxide) [21,22] and polyvinylidene-fluoride-co-hexafluoropropylene-based (PVDF-HFP) membranes in prior studies [23,24]. The composite membrane demonstrates stable performance with only a slight increase in overpotential during 2300 h of testing. This finding indicates that the composite membrane reported here provides two key advantages over traditional materials by: (i) mitigating the electrochemical reduction of Nafion in contact with Na; and (ii) increasing mechanical strength, which is critical for the targeted end-use application in NARFBs.

## 4. Conclusions

A Na^+^-Nafion-Celgard composite membrane was developed, which shows promising performance for NARFB applications. More specifically, Na^+^-Nafion was embedded in a porous Celgard support using a scalable solution casting procedure. The composite membrane demonstrates a higher room temperature storage modulus than Celgard and Na^+^-Nafion separately while maintaining high Na^+^ conductivity (0.26 mS/cm at 20 °C). The composite membrane also exhibits improved electrochemical stability compared to Na^+^-Nafion in both a Na_2_S_8_/Na full cell and Na symmetric cell configuration. The permeability of the redox active species, Na_2_S_8_, through the thin Na^+^-Nafion selective layer of the composite membrane is higher than Na^+^-Nafion (1.4 × 10^−7^ and 3.1 × 10^−8^ cm^2^/s, respectively) and could be improved through optimized membrane design and fabrication. Overall, the composite membrane approach demonstrates that a bilayer design with an electrochemically inert layer combined with a thin selective layer is a viable path toward obtaining high-performance membranes for RFBs containing an alkali metal anode.

## Figures and Tables

**Table 1:** Properties of the various membranes.

Sample	Na_2_S_8_ Permeability (cm^2^/s)	Conductivity (mS/cm, 20 °C)	Area Specific Resistance (Ω cm^2^, 20 °C)	Storage Modulus (MPa, 25 °C)
Celgard	2.2 × 10^−6^	0.44	5.2	584
Na^+^-Nafion	3.1 × 10^−8^	0.17	19.5	318
Composite	1.4 × 10^−7^	0.26	23.7	935

## Data Availability

Data are available upon request.
